# Long Non-coding RNAs and Circular RNAs: Insights Into Microglia and Astrocyte Mediated Neurological Diseases

**DOI:** 10.3389/fnmol.2021.745066

**Published:** 2021-10-05

**Authors:** Miaomiao Chen, Xingning Lai, Xifeng Wang, Jun Ying, Lieliang Zhang, Bin Zhou, Xing Liu, Jing Zhang, Gen Wei, Fuzhou Hua

**Affiliations:** ^1^Department of Anesthesiology, The Second Affiliated Hospital of Nanchang University, Nanchang, China; ^2^Key Laboratory of Anesthesiology of Jiangxi Province, Nanchang, China; ^3^First Clinical Medical College, Nanchang University, Nanchang, China; ^4^Department of Anesthesiology, West China Hospital, Sichuan University, Chengdu, China; ^5^Department of Anesthesiology, the First Affiliated Hospital of Nanchang University, Nanchang, China

**Keywords:** lncRNA, circRNA, microglia, astrocyte, neurological diseases

## Abstract

Microglia and astrocytes maintain tissue homeostasis in the nervous system. Both microglia and astrocytes have pro-inflammatory phenotype and anti-inflammatory phenotype. Activated microglia and activated astrocytes can contribute to several neurological diseases. Long non-coding RNAs (lncRNAs) and circular RNAs (circRNAs), two groups of non-coding RNAs (ncRNAs), can function as competing endogenous RNAs (ceRNAs) to impair the microRNA (miRNA) inhibition on targeted messenger RNAs (mRNAs). LncRNAs and circRNAs are involved in various neurological disorders. In this review, we summarized that lncRNAs and circRNAs participate in microglia dysfunction, astrocyte dysfunction, neuron damage, and inflammation. Thereby, lncRNAs and circRNAs can positively or negatively regulate neurological diseases, including spinal cord injury (SCI), traumatic brain injury (TBI), ischemia-reperfusion injury (IRI), stroke, neuropathic pain, epilepsy, Parkinson’s disease (PD), multiple sclerosis (MS), and Alzheimer’s disease (AD). Besides, we also found a lncRNA/circRNA-miRNA-mRNA regulatory network in microglia and astrocyte mediated neurological diseases. Through this review, we hope to cast light on the regulatory mechanisms of lncRNAs and circRNAs in microglia and astrocyte mediated neurological diseases and provide new insights for neurological disease treatment.

## Introduction

Both microglia and astrocytes are glial cells in the central nervous system (Kwon and Koh, 2020). Microglia are resident immune cells and the first responders to nervous system changes. Astrocytes can modulate cerebral blood flow (Greenhalgh et al., 2020). And both of them can also promote neurotransmission, synapse formation, and synapse pruning (Vainchtein and Molofsky, 2020). In response to pathological conditions, microglia and astrocytes are activated. Activated microglia and activated astrocytes lose their homeostatic functions and involve in neuroinflammation in the central nervous system (Leng and Edison, 2021). Increasing evidence investigates that the dysfunctions of microglia and astrocytes are beneficial for various neurological diseases, like central nervous system injury and neurodegenerative disorders (Gao et al., 2013; Jha et al., 2019; Pietrowski et al., 2021). Targeting microglia and astrocytes could be a promising approach for the treatment of neurological diseases (Peterson and Binder, 2020; Huang X. et al., 2017).

Long non-coding RNA (lncRNA) and circular RNA (circRNA) are two types of non-coding RNAs (ncRNAs) without the potential to encode proteins (Han T. S. et al., 2020). LncRNAs are transcripts with more than 200 nucleotides in length. LncRNAs include five different subtypes: sense lncRNA, antisense lncRNA, bidirectional lncRNA, intergenic lncRNA, and intronic lncRNA (Braga et al., 2020). LncRNAs regulate gene transcription and translation via interacting with the 3′untranslated region (3′UTR) of messenger RNA (mRNA) (Dykes and Emanueli, 2017). MicroRNAs (miRNAs) are small single-stranded RNAs. MiRNAs recognize miRNA response elements (MREs) on 3′UTR of mRNA and silence target genes through repressing mRNA translation or degrading the mRNA (Cheng et al., 2015; Salim et al., 2021). LncRNAs contain MREs and can use MREs to bind miRNAs. LncRNAs function as competing endogenous RNAs (ceRNAs) to sponge miRNAs and reduce miRNA activity, finally, impair the interaction between miRNAs and their targeted genes (Kalpachidou et al., 2020; Moreno-García et al., 2020). LncRNAs have been demonstrated to involve in epigenetic mediation and the modulation of transcription, translation, RNA metabolism, cell autophagy, cell apoptosis, etc. (Qian et al., 2019). In particular, it is suggested that lncRNAs can influence neural cell behaviors, including neurons, microglia, astrocytes, and oligodendrocytes (Shi et al., 2018). For example, lncRNA AK148321 can abrogate microglial neuroinflammation (Gao et al., 2021). LncRNAs are also related to the development of spinal cord injury (SCI), brain injury, and neurodegenerative diseases (Cuevas-Diaz Duran et al., 2019). CircRNAs have covalently closed loop structures without 5′ caps or 3′ Poly A tails. CircRNAs are stable in eukaryotic cells. CircRNAs can interact with RNA-binding proteins and modulate mRNA stability, and gene transcription. It is proved that endogenous circRNAs can be translated into proteins (Chen and Yang, 2015; Meng et al., 2017). Moreover, like lncRNAs, circRNAs also have MREs. CircRNAs also act as ceRNAs and inhibit miRNA repression on its targeted mRNA (Moreno-García et al., 2020). Many studies report that circRNAs can influence cell proliferation, autophagy, differentiation, and pyroptosis (Fu and Sun, 2021). For instance, one research indicated that circHIPK2 knockout can suppress astrocyte autophagy and astrocyte activation (Huang R. et al., 2017). CircRNAs also play pivotal roles in neurological diseases, including ischemic stroke and cerebral ischemia-reperfusion injury (IRI) (Yang et al., 2018).

In this review, we discussed the effects of both lncRNAs and circRNAs on microglia and astrocyte mediated neurological diseases. We also found a network of lncRNA/circRNA-miRNA-mRNA in neurological diseases. In addition, there are also targeted therapies for the treatment of neurological diseases.

## Microglia and Astrocytes in the Nervous System

### Microglia

As tissue-resident macrophages derived from progenitors in the embryonic yolk sac, microglia are parenchyma cells and constitute approximately 10% of central nervous system cells (Salter and Stevens, 2017). Under physiological conditions, microglial cells remain in a resting state. During embryonic and postnatal development, microglia can remove apoptotic neurons, support neuronal survival, guide vascularization, and regulate synaptic pruning. In the adult brain, microglial cells are necessary for adult neurogenesis, skill learning, memory formation, and normal behaviors (Kierdorf and Prinz, 2017). Under pathologic conditions, microglial cells undergo the transformation from the resting state to an activated state (Hanisch and Kettenmann, 2007). M1 microglia (classical activation) can release pro-inflammatory factors, such as tumor necrosis factor-α (TNF-α), interleukin-1 (IL-1), IL-6, IL-12, inducible nitric oxide synthase (iNOS). M2 microglia (alternative activation) can produce anti-inflammatory factors, including transforming growth factor-β, IL-4, and IL-10. M1 microglia can promote inflammation and delay tissue repair. While M2 microglia can attenuate inflammation and promote tissue regeneration (Hu et al., 2015). Plenty of works suggested that microglial dysfunction participates in several neurological disorders, including SCI, brain injury, depression, stroke, epilepsy, neuropathic pain, Parkinson’s disease (PD), multiple sclerosis (MS), and Alzheimer’s disease (AD) (Wolf et al., 2017; Araki et al., 2020; Tripathi et al., 2021).

### Astrocytes

Astrocytes are the most abundant glial cells within the central nervous system. Astrocytes can interact with brain endothelial cells to form the blood-brain barrier (Abbott et al., 2006). Besides, astrocytes are also essential for synaptic plasticity, memory consolidation, cognitive function, and neuronal survival (Santello et al., 2019). Under pathological conditions (e.g., stroke, infection, ischemia, cancer, etc.), astrocytes become activated (Pekny and Nilsson, 2005). Activated astrocytes can be classified into A1 astrocytes (pro-inflammatory phenotype) and A2 astrocytes (anti-inflammatory phenotype) (Liu L. R. et al., 2020). A2 astrocytes are helpful for neurons and can promote neuron survival and growth. While A1 astrocytes are neurotoxic and stimulate neuron death (Liddelow and Barres, 2017). Activated astrocytes are involved in various neurological diseases, like stroke, epilepsy, SCI, brain injury, and neurodegenerative diseases (Karthikeyan et al., 2016; Nutma et al., 2020).

## Long Non-Coding RNAs in Microglia Mediated Neurological Diseases

### Spinal Cord Injury

Spinal cord injury is one neurological disease with impairment of sensory and motor function. The SCI pathological process includes primary and secondary injury (Anjum et al., 2020). Primary SCI occurs owing to mechanical damage, for instance, compression or contusion in the spinal cord. Secondary SCI is caused by pathological changes after primary injury, which can result in inflammation, oxidative stress, glial scar formation, neuronal apoptosis, and neuronal necrosis (Anjum et al., 2020; Wang et al., 2020d). Recently, SCI models induced by lipopolysaccharide (LPS) are widely used to explore SCI pathogenesis and find new therapeutic medicine to treat SCI (Xiao et al., 2021).

MiR-382-5p expression is significantly induced by LPS in BV2 cells (mouse microglial cell lines). MiR-382-5p targets 3′UTR of Neuregulin-1 (Nrg1). MiR-382-5p deletion stimulated by its inhibitor can remarkably increase Nrg1 expression in BV2 cells (Xiang et al., 2021). LncRNA Ftx expression is markedly decreased in tissues collected from SCI mice and in LPS-stimulated BV2 cells. Ftx targets miR-382-5p and represses its expression. Besides, Ftx also competes with Nrg1 for miR-382-5p binding, resulting in suppressing miR-382-5p repression on Nrg1 expression (Xiang et al., 2021). Ftx over-expression increases Nrg1 expression levels through miR-382-5p. Increased Nrg1 attenuates the expressions of inflammation factors (e.g., iNOS, IL-6, TNF-α, and IL-1β) in LPS-treated BV2 cells. As a result, increased Ftx relieves microglia inflammation response through the miR-382-5p-Nrg1 axis (Xiang et al., 2021). In addition, this study also indicated that over-expressed Ftx can suppress inflammation response and improve functional recovery in the SCI mouse model (Xiang et al., 2021). Exosomes, which belong to extracellular vesicles (EVs), can be secreted by all cells and carry various materials, including proteins, lipids, mRNA, ncRNAs, etc. And exosomes derived from mesenchymal stem cells possess the potential for SCI treatment (Ren et al., 2020). It is reported that peroxisome proliferator-activated receptor γ (PPARγ) can suppress inflammatory phenotype in macrophages (Nelson et al., 2018). MiR-130b-3p can interact with 3′UTR of PPARγ and reduce its expression. LncGm37494 is reported to be abundant in exosomes originated from hypoxia-treated adipose-derived stem cells (ADSCs). Exosomal lncGm37494 from ADSCs can shift microglia polarization from M1 to M2 via decreasing miR-130b-3p and increasing PPARγ. Finally, exosomal lncGm37494 contributes to improving functional recovery following SCI in mice (Shao et al., 2020). In the SCI rat model, receptor-activity modifying proteins 3 (RAMP3) is up-regulated and miR-222-5p is decreased. MiR-222-5p can target RAMP3 3′UTR and inhibit RAMP3 expression. Enhanced RAMP3 expression or miR-222-5p inhibitor can promote cell apoptosis and increase TNF-α and IL-6 expressions in LPS-treated microglia (Cui et al., 2021). LncRNA lymphoid enhancer-binding factor 1 (LEF1) antisense RNA 1 (LEF1-AS1) is up-regulated in LPS-treated microglia cells that are collected from the central nervous system of normal rats. LEF1-AS1 acts as a ceRNA via sponging miR-222-5p. LEF1-AS1 small interfering RNA (siRNA) can augment miR-222-5p expression. Knocking down LEF1-AS1 dampens microglia apoptosis and promotes microglial cell viability through the miR-222-5p-RAMP3 axis. LEF1-AS1 silence can impede SCI progression in rats (Cui et al., 2021). Pyroptosis is a type of programmed cell death involved in neuroinflammation (Dai et al., 2019). And it is proved that inhibiting microglial pyroptosis can promote motor function recovery following SCI (Xu S. et al., 2020). F630028O10Rik levels are higher in the peripheral blood from SCI patients compared with normal people. F630028O10Rik can sponge miR-1231-5p to up-regulate Col1a1. Subsequently, increased Colla1 can enhance microglial pyroptosis through the activation of phosphoinositide 3-kinase (PI3K)/protein kinase B (AKT) pathway. Increased F630028O10Rik expression can indicate worse functional recovery after SCI (Xu S. et al., 2020). Smad ubiquitination regulatory factor 1 (Smurf1) is a downstream target of miR-27a and its expression can be attenuated by miR-27a. Smurf1 over-expression or miR-27a down-regulation can promote TNF-α and IL-6 secretion in LPS-treated microglia (Zhao Q. et al., 2020). LncRNA X-inactive specific transcript (XIST) expression is elevated in LPS-treated microglial cells and in injured rat spinal cord tissues. XIST sponges miR-27a and negatively mediates miR-27a expression. XIST deletion abrogates SCI-evoked microglia apoptosis and inflammatory injury through the miR-27a up-regulation and Smurf1 down-regulation. Moreover, the deficiency of XIST can relieve SCI (Zhao Q. et al., 2020). The expression of lncRNA metastasis-associated lung adenocarcinoma transcript 1 (MALAT1) is higher in SCI rats compared to normal rats. Over-expressed MALAT1 activates the IkappaB kinase β (IKKβ)/nuclear factor kappa B (NF-κB) signaling pathway by down-regulating miR-199b. Eventually, MALAT1 over-expression promotes pro-inflammatory cytokine production in LPS-treated microglial cells (Zhou et al., 2018). Zhou et al. (2018) also revealed that MALAT1 silence can alleviate SCI through inhibiting microglial inflammatory responses.

### Traumatic Brain Injury

Traumatic brain injury (TBI) is recognized as a primary reason for high morbidity and high disability around the world (Jamjoom et al., 2021). The primary injury of TBI occurs directly in response to mechanical forces, like contusion, hemorrhage, hypoxia, etc. The primary injury can trigger the secondary injury that relates to several biochemical alterations, such as inflammation, nerve cell damage, and cerebral edema, contributing to exacerbating TBI progression (Alam et al., 2020; Gasco et al., 2021). Tumor necrosis factor receptor-related factor 6 (TRAF6) is a target of miR-873-5p. LncRNA KCNQ1 overlapping transcript 1 (KCNQ1OT1) is up-regulated in the cerebral lesions of TBI mice. KCNQ1OT1 down-regulation can increase miR-873-5p expression. Reducing KCNQ1OT1 blocks microglial inflammation through the miR-873-5p-TRAF6 axis (Liu N. et al., 2021). Liu N. et al. (2021) revealed that KCNQ1OT1 depletion can mitigate TBI-induced injuries, including neurological deficits, neuron loss, brain edema, and blood-brain barrier damage (Liu N. et al., 2021). Many studies revealed that inhibiting microglial activation and neuroinflammation can hamper TBI development (Kumar et al., 2017). The E3 ubiquitin ligase Nrdp1 can interact with myeloid differentiation factor-88 adaptor protein (MYD88) and decrease MYD88 protein levels (Cheng et al., 2021). LncRNA HOX antisense intergenic RNA (HOTAIR) is up-regulated in the cerebral cortex of TBI mice and in LPS-treated BV2 cells. HOTAIR up-regulation attenuates the interaction between Nrdp1 and MYD88, thus, to increase MYD88 protein expression. HOTAIR knockout enhances Nrdp1-mediated the down-regulation of MYD88 to suppress microglial activation and microglia inflammatory factor (e.g., TNF-α, IL-1β, and IL-6) release, which can ultimately repress TBI (Cheng et al., 2021). Nod-like receptor protein 3 (NLRP3) is a target of miR-7a-5p. NLRP3 absence decreases iNOS expression, nitric oxide (NO) production, and inflammatory cytokine (e.g., IL-1β, IL-6, and TNF-α) expression (Meng et al., 2021). LncRNA maternally expressed gene 3 (MEG3) is over-expressed in the TBI cell model (LPS + ATP induced microglial cells that are obtained from normal mice). MEG3 targets miR-7a-5p and reduces its expression. MEG3 also functions as a ceRNA for miR-7a-5p and represses miR-7a-5p inhibition on NLRP3. Functionally, MEG3 up-regulation enhances microglial activation and inflammation through the miR-7a-5p-NLRP3 axis in the TBI *in vitro* model (Meng et al., 2021).

### Ischemia-Reperfusion Injury

More and more evidence revealed that recovery of blood flow in a short time after cerebral ischemia can lead to cerebral IRI (Pan et al., 2007). IRI gives rise to reactive oxygen species (ROS), calcium overload, neuron death, platelet activation, and inflammation, which can ultimately cause irreversible damage to the brain (Wu et al., 2010; Bai and Lyden, 2015). In research studies, middle cerebral artery occlusion (MCAO)/reperfusion (MCAO/R) treatment *in vivo* and oxygen-glucose deprivation (OGD)/reoxygenation (OGD/R) treatment *in vitro* are used to mimic cerebral IRI (Zheng et al., 2021). It is reported that up-regulated Krüppel-like factor 4 (KLF4) decreases M1 marker (CD86 and iNOS), TNF-α, and IL-1β levels, while increases M2 marker (CD206 and Arg1), IL-10, and IL-4 expressions in OGD/R-induced BV2 cells (Li T. et al., 2020). MEG3 is elevated in the brain tissues from MCAO/R mice and in OGD/R-induced BV2 cells. MEG3 binds to KLF4 and represses its protein progression. MEG3 absence enhances M2 polarization and reduces M1 polarization in microglial cells by targeting KLF4. Eventually, MEG3 knockdown decreases neuroinflammation to lessen cerebral IRI (Li T. et al., 2020).

Long non-coding RNA small nucleolar RNA host gene 4 (SNHG4) is remarkably down-regulated in blood and cerebrospinal fluid samples obtained from patients with acute cerebral infarction. Enhanced SNHG4 expression can facilitate microglia M2 polarization and promote anti-inflammatory factor release (Zhang S. et al., 2020). MiR-449c-5p can bind with the 3′UTR of signal transducers and activators of transcription 6 (STAT6). MiR-449c-5p inhibitor increases STAT6 expression. SNHG4 targets miR-449c-5p and inhibits its expression. Up-regulated SNHG4 activates the STAT6 signaling pathway by suppressing miR-449c-5p. Therefore, SNHG4 up-regulation attenuates microglial inflammatory responses and microglia-mediated neuronal damage during cerebral IRI (Zhang S. et al., 2020). LncRNA SNHG3 is elevated in OGD/R-stimulated BV2 cells. Interference with SNHG3 impedes microglial activation and dampens the release of pro-inflammatory factors (TNF-α and IL-6) through abrogating histone deacetylase 3 (HDAC3) expression (Huang D. et al., 2021). In transient MCAO mice, SNHG3 deletion weakens cerebral IRI (Huang D. et al., 2021). Diabetes mellitus (DM) is characterized by high sugar in the blood and can worsen cerebral IRI (Wang and Zhou, 2018). MALAT1 is highly expressed in cortical tissues of diabetic rats suffering the IRI and in HAPI cells (rat immortalized microglia cell line) undergoing high glucose and hypoxia-reoxygenation. Over-expressed MALAT1 enhances microglia inflammatory response through activating the MyD88/IRAK1 (IL-1 receptor-associated kinase 1)/TRAF6 signaling pathway. Finally, increased MALAT1 aggravates cerebral IRI in diabetic rats (Wang and Zhou, 2018). LncRNA nuclear enriched abundant transcript 1 (NEAT1) expresses highly in the blood of ischemic stroke patients and in OGD/R-induced BV2 cells (Ni et al., 2020). One research reported that NEAT1 can serve as a ceRNA for miR-224 and thus enhance IL-33 production and promote M2a polarization in macrophages. NEAT1 can also hinder A1 astrocyte activation (Liu D. et al., 2021). Ni et al. (2020) found that suppressing NEAT1 abrogates microglial M1 polarization to reduce neuronal apoptosis after cerebral IRI.

### Stroke

Stroke is a severe disorder with high rates of mortality and disability around the world. Stroke can be divided into ischemic stroke (major type) and hemorrhagic stroke (Xu et al., 2021). Ischemic stroke is mainly due to atherosclerosis in large vessels and atherosclerotic plaque ruptures. MCAO models in rodents and OGD *in vitro* models are usually used for experimental ischemic stroke researches (Sommer, 2017).

Malate dehydrogenase 2 (MDH2) binds to the mRNA of neutrophil chemoattractant C-X-C motif ligand 2 (CXCL2) and reduces CXCL2 mRNA levels (Chen J. et al., 2021). LncRNA U90926 is highly increased in microglia isolated from the ischemic hemispheres in C57BL/6J mice. U90926 interacts with MDH2 to impair the interaction between MDH2 and CXCL2 mRNA. Increased microglial U90926 enhances CXCL2 protein levels. Increased CXCL2 release from microglia can ultimately stimulate neutrophil infiltration, which can exacerbate brain injury during ischemic stroke. Once microglial U90926 is silenced, the neurological function is improved and infarct volume is diminished in ischemic stroke mice (Chen J. et al., 2021). LncRNA opa-interacting protein 5 antisense RNA 1 (OIP5-AS1) lowly expresses in the blood of stroke patients. It is shown that OIP5-AS1 down-regulation increases miR-186-5p levels (Chen Y. et al., 2021). Over-expressed miR-186-5p leads to microglia inflammation and oxidative stress via blocking the expression of C1q/tumor necrosis factor-related protein 3 (CTRP3), resulting in neuron damage during ischemic stroke. Further, OIP5-AS1 up-regulation lessens infarct volume and weakens neuron apoptosis and loss in rats with ischemic stroke (Chen Y. et al., 2021). One research demonstrated that lincRNA-EPS can be loaded in leukosomes (biomimetic vesicles). And these leukosomes carrying lincRNA-EPS can target the ischemic area and co-localize with microglia in ischemic stroke mice (Zhang B. et al., 2020). LincRNA-EPS from leukosomes impedes microglia activation and microglia infiltration *in vivo*. In addition, leukosomes-loaded lincRNA-EPS can also accelerate neuron regeneration via hindering microglia inflammation in cerebral infarction (Zhang B. et al., 2020). It is said that sirtuin1 activation can abolish NF-κB signaling (Kauppinen et al., 2013). MiR-425-5p can target sirtuin1. In brain tissues of MCAO mice, SNHG8 expression is down-regulated. MiR-425-5p can share binding sites with SNHG8 and sirtuin1. SNHG8 functions as a ceRNA for miR-425-5p and inhibits miR-425-5p effects on sirtuin1 (Tian et al., 2021). SNHG8 over-expression can attenuate miR-425-5p to increase sirtuin1 expression and suppress NF-κB phosphorylation. SNHG8 amplification suppresses microglial activation and inflammation through the miR-425-5p/sirtuin1/NF-κB pathway, resulting in diminishing brain microvascular endothelial cell (BMEC) damage. Furthermore, after ischemic stroke, over-expressed SNHG8 dampens neuron damage, cerebral edema, and neurological function loss in mice (Tian et al., 2021). H19 levels are significantly raised in the plasma isolated from stroke patients compared to normal people. Repressing H19 reduces HDAC1 to facilitate microglial polarization from M1 to M2. H19 inhibition promotes cerebral IL-10 production and decreases cerebral TNF-α and IL-1β levels in ischemic mice. Therefore, H19 reduction can inhibit neuroinflammation in ischemic stroke (Wang et al., 2017). In ischemic stroke mice, H19 siRNA decreases cerebral ischemic injury and attenuates neurological deficits. Moreover, H19 would be a promising biomarker to diagnose ischemic stroke (Wang et al., 2017). LncRNA Nespas is greatly expressed in ischemic cerebral tissues of MCAO mice and in BV2 cells subjected to OGD. High Nespas expression hinders transforming growth factor-beta-activated kinase 1 (TAK1) activation via abolishing the interaction between tripartite motif 8 (TRIM8) and TAK1 (Deng et al., 2019). Over-expressed Nespas limits NF-κB activation through TAK1, which can restrain microglia death and neuroinflammation in the ischemic microenvironment. Moreover, Nespas silence in MCAO mice deteriorates ischemic brain injury (Deng et al., 2019). LncRNA 1810034E14Rik declines markedly in microglial cells after the OGD exposure. Elevated 1810034E14Rik expression impedes microglia activation and microglia inflammation *in vitro* and *in vivo* (Zhang et al., 2019b). In addition, up-regulated 1810034E14Rik can abrogate neuronal damage caused by OGD-treated microglia. Further, enhanced 1810034E14Rik expression promotes motor function recovery and decreases infarct volumes in MCAO mice (Zhang et al., 2019b). SNHG14 and cytosolic phospholipase A2 (cPLA2) group IVA (PLA2G4A) are notably up-regulated in ischemic cerebral tissues and in OGD-treated BV2 cells. MiR-145-5p is down-regulated in MACO mice. PLA2G4A is a downstream target of miR-145-5p. The miR-145-5p inhibitor can increase TNF-α, and nitrate levels in OGD-treated BV2 cells (Qi et al., 2017). SNHG14 elevates PLA2G4A expression and abrogates miR-145-5p expression. Functionally, SNHG14 can facilitate microglia activation and microglia-mediated neuron apoptosis through the miR-145-5p-PLA2G4A axis in response to ischemic stroke conditions. Further, highly expressed SNHG14 can act as an induction effector of cerebral infarction severity (Qi et al., 2017). MiR-96-5p abrogates NF-κB signaling and TNF-α production via suppressing IKKβ expression in OGD-stimulated BV2 cells (Zhang M. et al., 2021). LncRNA XIST is highly expressed in BV2 cells exposed to OGD treatment. XIST serves as a sponge for miR-96-5p to antagonize inhibitory effects of miR-96-5p on the TNF-α expression. XIST up-regulation increases TNF-α expression through the miR-96-5p-IKKβ-NF-κB axis (Zhang M. et al., 2021). Moreover, TNF-α stimulation can increase XIST mRNA levels in BV2 cells. The XIST-TNF-α feedback enhances the pro-inflammatory polarization of microglial cells, leading to exacerbating cerebral neuron apoptosis in cerebral infarction (Zhang M. et al., 2021).

### Neuropathic Pain

Accumulating evidence reveals that neuropathic pain develops owing to the diseases or damaging stimuli in the nervous system. Neuropathic pain includes postherpetic neuralgia, trigeminal neuralgia, and nerve injury pain. Patients with neuropathic pain usually suffer from allodynia and hyperalgesia (Jensen and Finnerup, 2014). It is investigated that enhancer of zeste homolog 2 (EZH2), one RNA-binding protein of Lncenc1, can promote histone methylation in the promoter of brain-specific angiogenesis inhibitor 1 (BAI1) and decrease BAI1 expression (Zhang Z. et al., 2021). After nerve injury, lncRNA embryonic stem cells expressed 1 (Lncenc1) is notably increased in dorsal root ganglion in mice. Increased Lncenc1 enhances EZH2 expression to down-regulate BAI1, which can accelerate microglia activation and microglial inflammatory response. Decreasing Lncenc1 can impede neuropathic pain development in rats (Zhang Z. et al., 2021).

### Epilepsy

Epilepsy is a neurological disorder characterized by a persistent predisposition to produce epileptic seizures (Fisher et al., 2014). Epilepsy includes focal epilepsy, generalized epilepsy, combined generalized and focal epilepsy, and unknown epilepsy (the patient has epilepsy but the clinician cannot determine if the epilepsy is focal epilepsy or generalized epilepsy) (Scheffer et al., 2017). Temporal lobe epilepsy is the most common form of focal epilepsy. And patients with temporal lobe epilepsy usually experience hippocampal sclerosis (Lévesque and Avoli, 2013). Epilepsy can be caused by genetic mutations, stroke, TBI, infection, cancers, etc. Although there are many anti-seizure drugs, approximately one-third of patients can’t achieve seizure control (Devinsky et al., 2018). Recently, rodents with spontaneous seizures that mimic human epilepsy can be used to study epilepsy. For example, the kainic acid (KA)-induced epilepsy model is widely used to explore pathogenetic mechanisms of temporal lobe epilepsy (Lévesque and Avoli, 2013). H19 expression is notably higher in the hippocampus in epileptic rats compared to normal rats (Han C. L. et al., 2020). Han C. L. et al. (2018) showed that H19 can stimulate hippocampal microglia and astrocyte activation through the Janus kinase (JAK)/STAT3 signaling pathway in a KA-induced rat model of temporal lobe epilepsy. Another research investigated that H19 can bind to miRNA let-7b and restrain its expression. Increased H19 promotes STAT3 mRNA expression levels via sponging let-7b. Subsequently, up-regulated STAT3 results in microglia and astrocyte activation during epileptogenesis. Eventually, the activation of both microglia and astrocytes can aggravate epileptic seizures in rats undergoing temporal lobe epilepsy induced by KA (Han C. L. et al., 2020). Feng et al. (2020) revealed that up-regulated 26S proteasome non-ATPase regulatory subunit 11 (Psmd11) can not only inactivate microglia and astrocytes but also abrogate neuron injury and inflammation in the epileptic hippocampus in mice. In addition, Psmd11 is a downstream target of miR-490-3p (Feng et al., 2020). LncRNA Peg13 is lowly expressed in epileptic hippocampal tissues from mice. Peg13 acts as a ceRNA for miR-490-3p. Peg13 competitively binds with miR-490-3p to elevate Psmd11 expression. Ultimately, in epileptic mice, high Peg13 expression suppresses epilepsy progression (Feng et al., 2020).

### Depression

Depression is a mood disorder affecting human beings at any age. Depressed people usually experience persistent sadness, decreased interest, loss of pleasure, diminished or increased sleep, etc. Childhood maltreatment (e.g., abuse, loss, and neglect) and stressful life can increase the risk of depression (Malhi and Mann, 2018). It is indicated that 2007 lncRNAs were differentially expressed in peripheral blood of major depressive disorder (MDD) patients compared to that in normal people, including 1,556 up-regulated lncRNAs and 441 down-regulated lncRNAs (Liu et al., 2014). LncRNAs related to cognitive function, synaptic plasticity, and psychiatry diseases (e.g., schizophrenia) potentially contribute to MDD (Huang X. et al., 2017). LncRNA uc.80- is down-regulated in the hippocampus obtained from depressed rats induced by chronic unpredictable mild stress (CUMS) regiment. Over-expressed uc.80- inhibits microglia M1 polarization and facilitates microglia M2 polarization. In addition, uc.80- up-regulation can attenuate neuron apoptosis mediated by microglia under inflammation exposure. Functionally, uc.80- over-expression ameliorates depression via enhancing M2 polarization of microglial cells in CUMS-treated rats (Gu et al., 2020).

### Parkinson’s Disease

Parkinson’s disease, the second most common neurodegenerative disorder after AD, is characterized by the loss of dopaminergic neurons within the substantia nigra pars compacta (Jankovic and Tan, 2020). The clinical features in PD patients include classical motor symptoms, cognitive impairment, pain, psychiatric symptoms, etc. (Kalia and Lang, 2015). LncRNA HOXA11-AS is detected to be up-regulated in the substantia nigra area in PD mice. HOXA11-AS over-expression can elevate the levels of inflammatory factors, including, IL-1β, IL-18, IL-6, and TNF-α in LPS-mediated BV2 cells (Cao et al., 2021). HOXA11-AS can target miR-124-3p that can target follistatin-like 1 (FSTL1). Down-regulated HOXA11-AS enhances miR-124-3p expression to inhibit FSTL1 and NF-κB expressions. The inactivation of the FSTL1/NF-κB pathway can dampen neuron damage and inhibit microglial inflammation. Ultimately, HOXA11-AS down-regulation mitigates PD progression in PD mice (Cao et al., 2021). MALAT1 highly expresses in 1-Methyl-4-phenyl-1,2,3,6-tetrahydropyridine (MPTP)-induced PD mouse brain tissues and in and in LPS/ATP-induced BV2 cells. MALAT1 can recruit the EZH2 to the promoter of nuclear factor (erythroid-derived 2)-like-2 factor (NRF2) and then inhibit NRF2 expression. Repressing NRF2 decreases IL-1β, IL-18, and TNF-α *in vitro* (Cai et al., 2020b). MALAT1 deficiency can abrogate inflammasome activation and ROS generation through NRF2 in microglia. The MALAT1 elimination in PD mice blocks neuron damage by reducing microglia inflammation (Cai et al., 2020b). LincRNA-p21 is shown to be induced by p53 in LPS-treated BV2 cells. LincRNA-p21 acts as a ceRNA for the miR-181 family (miR-181a, miR-181b, miR-181c, and miR-181d), thus protecting protein kinase C δ (PKC-δ) from miR-181 family-regulated suppression. Increased lincRNA-p21 can augment PKC-δ by competitively binding with the miR-181 family (Ye et al., 2018). In turn, increased PKC-δ augments the levels of p53 and lincRNA-p21. The lincRNA-p21 mediated regulatory loop with miR-181 family and PKC-δ can contribute to microglia activation and inflammation. Besides, increased lincRNA-p21 also accelerates neuron degeneration caused by microglia inflammation in PD mice (Ye et al., 2018). The expression levels of SNHG1 are increased in LPS-induced BV2 cells and in brain tissues in PD patients. SNHG1 is a ceRNA for miR-7. Knocking down SNHG1 can increase miR-7 to down-regulate NLRP3 expression. Then decreased NLRP3 reduces microglial activation and inflammation. Eventually, SNHG1 knockdown attenuates neuronal apoptosis in PD mice (Cao et al., 2018). It is shown that miR-223-3p can target NLRP3 to impair its expression. Over-expressed miR-223-3p can impede NLRP3 inflammasome activation and decrease inflammatory cytokine expressions, including IL-1β, IL-6, and TNF-α (Xu W. et al., 2020). LncRNA growth arrest-specific 5 (GAS5) is significantly over-expressed in PD mice. GAS5 can repress miR-223-3p expression and abrogates miR-223-3p suppression on NLRP3. Elevated GAS5 can trigger microglia inflammation via the miR-223-3p-NLRP3 axis. Silencing GAS5 can hamper behavioral impairments in the PD mouse model (Xu W. et al., 2020).

### Multiple Sclerosis

Multiple sclerosis is a chronic inflammatory disease in the central nervous system. The MS pathological process consists of multifocal inflammation, demyelination, reactive gliosis, and axonal degeneration (Baecher-Allan et al., 2018). GAS5 is significantly increased in microglia from experimental autoimmune encephalomyelitis (EAE) mice (an animal MS model). Up-regulated GAS5 attenuates the expression of interferon-regulatory factor 4 (IRF4) via binding with EZH2, which can impede microglia M2 polarization. Besides, enhanced microglial GAS5 can exacerbate myelin loss in EAE mice (Sun et al., 2017).

## Long Non-Coding RNAs in Astrocyte Mediated Neurological Diseases

### Spinal Cord Injury

Long non-coding RNA SNHG5 expression is increased in the astrocytes and microglia isolated from SCI rats. High SNHG5 expression elevates KLF4 expression. Enhanced SNHG5 expression improves cell viability and hampers apoptosis through KLF4 in astrocytes and microglia. In SCI rats, SNHG5 up-regulation promotes SCI progression (Jiang and Zhang, 2018).

### Traumatic Brain Injury

Extracellular vesicles are cargo-carrying vesicles that can affect cell communication (Teng and Fussenegger, 2020). MiR-195 can target nucleotide-binding leucine-rich repeat containing family member X1 (NLRX1). LncRNA nuclear transcription factor NF-κB interacting lncRNA (NKILA) can be transferred from astrocytes into neurons through EVs. NKILA functions as a ceRNA for miR-195. NKILA competitively binds to miR-195 and counteracts miR-195 inhibitory effect on NLRX1 (He et al., 2021). NKILA from astrocyte-derived EVs diminishes miR-195 to augment NLRX1 expression. Subsequently, increased NLRX1 enhances proliferation and declines apoptosis in injured neurons. Moreover, in TBI mice, NKILA-enriched EVs from astrocytes accelerate brain recovery (He et al., 2021). It is reported that astrocyte swelling is one of the early consequences of TBI-induced brain edema. When astrocyte swelling occurs, the astrocyte volume is increased (Zhang et al., 2019d). MALAT1 expression is decreased in the edematous brain after TBI. Over-expressed MALAT1 abrogates astrocyte swelling through attenuating aquaporin 4 (AQP4), NF-κB, and IL-6 expressions in response to trauma. This study suggests the negative relationship between MALAT1 and TBI-related brain edema (Zhang et al., 2019d).

### Ischemia-Reperfusion Injury

Metastasis-associated lung adenocarcinoma transcript 1 is up-regulated in OGD/R-treated MA-C cells (astrocyte cells). AQP4 is a downstream target of miR-145. MALAT1 suppression can reduce AQP4 via stimulating miR-145 expression. AQP4 silence attenuates astrocyte damage under ischemia-reperfusion conditions *in vitro* (Wang et al., 2020c). In MACO/R mice, MALAT1 deficiency decreases cell apoptosis and alleviates neurological deficits. This research revealed that MALAT1 inhibition can attenuate cerebral IRI through the miR-145-AQP4 axis (Wang et al., 2020c). LncRNA taurine upregulated gene 1 (TUG1) is highly expressed in OGD/R-treated MA-C cells. MiR-142-3p binds to TUG1 3′UTR. TUG1 is a miRNA sponge of miR-142-3p (Li et al., 2021). TUG1 down-regulation increases astrocyte cell viability and hinders astrocyte cell apoptosis via increasing miR-142-3p expression. This study revealed a positive relationship between TUG1 and cerebral IRI (Li et al., 2021).

### Neuropathic Pain

It is investigated that SNHG5 levels are remarkably increased in mouse spinal cord after spinal nerve ligation (SNL). In SNL mice, miR-154-5p elevation restrains CXCL13 protein levels, and inhibiting SNHG5 augments miR-154-5p levels (Chen M. et al., 2020). Knocking down SNHG5 dampens astrocyte activation and microglia activation via the miR-154-5p-CXCL13 axis. Repressing astrocyte activation and microglia activation can lessen neuropathic pain in SNL mice. This study revealed that SNHG5 deficiency alleviates neuropathic pain through the miR-154-5p-CXCL13 axis (Chen M. et al., 2020).

### Epilepsy

It is said that adenosine deficiency is a hallmark of epilepsy. And therapeutic adenosine augmentation can be a promising approach for epileptic seizures (Boison, 2012). LncRNA cancer susceptibility candidate 2 (CASC2) is lowly expressed in the hippocampus in epileptic rats. Elevated CASC2 hinders astrocyte activation and restrains astrocyte adenosine metabolism via triggering phosphatase and tensin homolog (PTEN) expression during epilepsy. Increased CASC2 can also repress epilepsy seizures in epileptic rats (Zhu et al., 2020). LncRNA urothelial cancer-associated 1 (UCA1) expression is down-regulated in the hippocampus of temporal lobe epilepsy rats. UCA1 over-expression abrogates astrocyte activation and suppresses the expression of astrocyte glutamate aspartate transporter (GLAST) (one factor related to epilepsy) *in vivo*. And UCA1 up-regulation reduces the frequency of epilepsy seizures and promotes learning and memory in temporal lobe epilepsy rats (Wang et al., 2020b). Yu et al. (2020) revealed that up-regulated UCA1 can enhance myocyte enhancer factor 2C (MEF2C) expression in IL-1β-treated CTX-TNA2 (the epilepsy cell models). MEF2C declines inflammatory effector [e.g., IL-6, TNF-α, and cyclooxygenase 2 (COX-2)] expression via blocking NF-κB signal in IL-1β-treated astrocytes. Increased UCA1 ultimately attenuates astrocyte inflammation through MEF2C/NF-κB pathway, which can hinder epilepsy progression (Yu et al., 2020). It is indicated that miR-129-5p can diminish Notch1 mRNA levels. LncRNA NEAT1 expression levels are elevated in the temporal lobe tissues in epilepsy patients compared to normal people. NEAT1 can target miR-129-5p and inhibit its expression (Wan and Yang, 2020). Increased NEAT1 activates the Notch1 signaling pathway by suppressing miR-129-5p. Finally, NEAT1 up-regulation can enhance astrocyte inflammatory responses in IL-1β-induced epilepsy cell models (Wan and Yang, 2020). In the brain, the brain-derived neurotrophic factor (BDNF) is essential for plasticity, neuronal survival, dendritic branching, etc. (Lima Giacobbo et al., 2019). LncRNA plasmacytoma variant translocation 1 (PVT1) is elevated in hippocampus tissues collected from epileptic rats. PVT1 down-regulation attenuates the Wnt signaling pathway, which can boost BDNF expression, impede neuronal loss, repress astrocyte activation, and decrease pro-inflammatory cytokine (e.g., TNF-α, IL-1β, and IL-6) expressions. PVT1 silence in epileptic rats also promotes spatial memory and learning (Zhao et al., 2019).

### Depression

Postpartum depression (PPD) remains one of the most common complications caused by childbirth. Stress and adverse life events can increase the risk of PPD (Payne and Maguire, 2019). The hormone-simulated pregnancy-induced PPD mouse model is used to explore the cellular mechanisms of PPD. It is indicated that oxytocin receptors play protective roles in PPD (Zhu and Tang, 2020). LncRNA Gm14205 is noticed to be up-regulated in the hippocampus in PPD mice. Up-regulated Gm14205 decreases oxytocin receptors, resulting in activating the NLRP3 inflammasome in hippocampal astrocytes. This study suggests a positive relationship between Gm14205 and PPD progression (Zhu and Tang, 2020).

### Multiple Sclerosis

Long non-coding RNA Gm13568 expression is stimulated in astrocytes after IL-9 treatment. Gm13568 interacts with CREB-binding protein (CBP)/P300 to facilitate the expression and activation of Notch1 (Liu X. et al., 2021). The activated Notch1/STAT3 pathway contributes to the secretion of inflammatory cytokines (e.g., IL-6, TNF-α, and interferon-inducible protein-10) in activated astrocytes. As a result, Gm13568 promotes astrocyte inflammation to worsen the EAE process (Liu X. et al., 2021).

### Alzheimer’s Disease

Alzheimer’s disease, a frequent neurodegenerative disorder, is the leading cause of dementia among elderly individuals. AD patients usually suffer from memory impairment, executive dysfunction, language problems, etc. (Scheltens et al., 2016). MEG3 expression is declined in the hippocampus tissues in AD rats. Up-regulated MEG3 suppresses astrocyte activation and neuronal damage via abrogating PI3K/AKT signaling pathway. Moreover, increased MEG3 can relieve cognitive impairment in AD rats (Yi et al., 2019).

## Circular RNAs in Microglia and Astrocyte Mediated Neurological Diseases

### Spinal Cord Injury

It is reported that treating astrocytes with TNF-α *in vitro* can induce the inflammatory response after SCI. Elevated miR-488 attenuates inflammatory cytokine secretion in TNF-α-treated astrocytes. Chemokine CC motif ligand 2 (CCL2) is a downstream target of miR-488 (Chen et al., 2022). CircPrkcsh is up-regulated in mouse injured spinal cords and in TNF-α-treated astrocytes. CircPrkcsh serves as a miRNA sponge for miR-488. Silencing circPrkcsh decreases CCL2 expression via miR-488 up-regulation (Chen et al., 2022). Chen et al. (2022) revealed that circPrkcsh knockout represses inflammatory response after SCI through the miR-488-CCL2 axis.

### Ischemia-Reperfusion Injury

Neural stem cells (NSCs) can differentiate into neurons and astrocytes. The NSCs are important for brain tissue regeneration and tissue repair after injury (Gage and Temple, 2013). The expression of circRNA TTC3 (circTTC3) is up-regulated in MCAO/R mice. CircTTC3 can sponge miR-372-3p that targets Toll-like receptor 4 (TLR4). Depleting circTTC3 suppresses OGD-induced astrocyte injury and promotes NSC proliferation and differentiation via the miR-372-3p-TLR4 axis. The circTTC3 knockdown in MCAO/R mice can alleviate cerebral IRI (Yang et al., 2021).

### Stroke

The expression levels of circHECTD1 are over-expressed in the plasma collected from acute ischemic stroke patients. TCDD inducible poly (ADP-ribose) polymerase (TIPARP) is a downstream target of miR-142. CircHECTD1 can bind to miR-142 and function as a miR-142 sponge to inhibit miR-142 repression on TIPARP. Down-regulating circHECTD1 eliminates TIPARP through miR-142 (Han B. et al., 2018). Knockdown of circHECTD1 can attenuate astrocyte autophagy via the miR-142-TIPARP axis, resulting in blocking astrocyte activation. Decreased circHECTD1 can lessen cerebral ischemic stroke injury in MCAO mice (Han B. et al., 2018). This research also revealed that circHECTD1 could be a promising biomarker for acute ischemic stroke diagnoses (Han B. et al., 2018). CircSHOC2 is found to be abundant in exosomes derived from ischemic-preconditioned astrocytes. MiR-7670-3p can bind to sirtuin 1 (SIRT1) 3′UTR. CircSHOC2 serves as a miRNA sponge for miR-7670-3p and attenuates miR-7670-3p inhibitory effects on SIRT1 expression (Chen W. et al., 2020). Exosomal circSHOC2 from astrocytes can abrogate neuron apoptosis and neuron damage through the miR-7670-3p-SIRT1 axis. Finally, exosomal circSHOC2 ameliorates ischemic stroke-associated brain injuries (Chen W. et al., 2020).

### Epilepsy

CircHivep2 is shown to be notably diminished in KA-treated BV2 cells and in hippocampus tissues of epilepsy mice. Suppressor of cytokine signaling-2 (SOCS2) is a target of miR-181a-5p. CircHivep2 binds to and sponges miR-181a-5p. CircHivep2 can decline miR-181a-5p to increase SOCS2 in KA-treated BV2 cells (Xiaoying et al., 2020). CircHivep2 elevation can attenuate microglia activation and microglia inflammation via the miR-181a-5p-SOCS2 axis. Over-expressed circHivep2 can ameliorate epileptic seizures in epilepsy mice (Xiaoying et al., 2020). CircIgf1r is up-regulated in the brain tissues in mice with status epilepticus. Inhibiting circIgf1r drives astrocyte A2 polarization via promoting autophagy. Therefore, circIgf1r inhibition reduces astrocyte-associated neuronal toxicity in the epilepsy cell model. Moreover, circIgf1r deficiency in epilepsy mice can impede neuronal damage and dampen epileptic discharge (Shao et al., 2021).

### Depression

CircSTAG1 is significantly declined in the plasma and whole blood in depressed patients. In astrocytes, over-expressed circSTAG1 increases the demethylase alkB homolog 5 (ALKBH5) levels in the cell cytoplasm and reduces ALKBH5 translocation into the cell nucleus (Huang et al., 2020). Thus, circSTAG1 over-expression facilitates the methylation of fatty acid amide hydrolase (FAAH) mRNA and inhibits FAAH expression. Increased circSTAG1 ultimately hampers astrocyte dysfunction and thereby hinders depressive-like behaviors in mice with MDD (Huang et al., 2020). In the plasma and hippocampi of depressed mice, circHIPK2 expression levels are increased (Zhang et al., 2019c). One study indicated that circHIPK2 deficiency can inhibit astrocyte activation via reducing autophagy (Huang R. et al., 2017). Zhang et al. revealed that circHIPK2 expression can be attenuated by the transplantation of the NLRP3-deficient gut microbiota in depressed mice. Suppressing circHIPK2 can block astrocyte dysfunction and therefore reduce depression in mice (Zhang et al., 2019c). It is shown that increased HECT domain E3 ubiquitin protein ligase 1 (HECTD1) can reduce 90-kDa heat shock protein (HSP90) expression via ubiquitination. MiR-9 can bind with the 3′UTR of HECTD1 and repress HECTD1 expression (Zhang Y. et al., 2020). CircRNA DYM (circDYM) expression is down-regulated in the peripheral blood in patients with MDD CircDYM functions as a molecular sponge to interfere with miR-9 activity, leading to increased HECTD1. CircDYM over-expression inhibits HSP90 expression through the miR-9-HECTD1 axis, which can reduce microglial activation. Therefore, increased circDYM restrains depression in depressed mice (Zhang Y. et al., 2020).

### Alzheimer’s Disease

The circNF1-419 expression levels are up-regulated in senescent astrocytes. Up-regulated circNF1-419 promotes autophagy in astrocytes *in vitro* (Diling et al., 2019). In AD mice, astroglial circNF1-419 over-expression enhances autophagy via binding to Dynamin-1 and Adaptor protein 2 B1 proteins. Eventually, circNF1-419 up-regulation leads to repressing senile dementia in AD mice (Diling et al., 2019).

### Targeted Therapy

Long non-coding RNAs and circular RNAs participate in various neurological diseases, suggesting their potential to act as therapeutic targets. Bexarotene, which is a selective agonist of retinoid X receptor, can suppress TBI progression in mice (Zhong et al., 2017a). Bexarotene can stimulate NEAT1 expression to inhibit neuron apoptosis and microglial inflammation, resulting in promoting motor and cognitive function recovery in TBI mice (Zhong et al., 2017b). Curcumin, an anti-inflammatory agent, can be delivered into microglial cells via exosomes. Exosomal curcumin can repress LPS-induced brain inflammation and myelin oligodendrocyte glycoprotein (MOG)-induced EAE development in mice (Zhuang et al., 2011). Moreover, one research suggested that curcumin can increase GAS5 expression to alleviate post-stroke depression in rats (Cai et al., 2020a). Berberine belongs to traditional Chinese medicine. Berberine can elevate circHDAC9 expression in human neurons. Elevated circHDAC9 protects human neuron cells from 42-residue β-amyloid (Aβ42)-triggered neuronal damage via sponging miR-142-5p (Zhang N. et al., 2020). As mentioned above, targeting lncRNAs and circRNAs will be a promising strategy for the treatment of neurological diseases.

### Dexmedetomidine

Dexmedetomidine (DEX), an anesthetic adjuvant, has various advantages in clinical applications, including hemodynamic stability, the ability to promote postoperative recognition, sedative and analgesic effects (Zhao Y. et al., 2020). DEX reduces high-mobility group box 1 (HMGB1) via eliminating SNHG14, resulting in attenuating microglial activation. Subsequently, DEX restrains neurological deficits induced by IRI *in vivo* (Ta Na et al., 2020). Deng et al. indicated that DEX can increase lncRNA LOC102546895 levels in hippocampus tissues in rats undergoing postoperative cognitive dysfunction (POCD). LOC102546895 promotes the expression of Neuronal Per-Arnt-Sim domain protein 4 (Npas4) (one protective neuronal transcription factor), impedes proliferation, and enhances apoptosis in microglial cells (Deng et al., 2020). DEX treatment hampers inflammatory response and neuron injury in the hippocampus. DEX can also ameliorate cognitive deficits in POCD rats (Deng et al., 2020).

### Valproate

Valproate is an eight-carbon branched-chain fatty acid and possesses anticonvulsant properties. Valproate is approved for the management of epilepsy, bipolar disorders, and migraine (Löscher, 1999). Li et al. reported that valproate can decline lncRNA RMRP levels to activate PI3K/AKT pathway, which can promote proliferation and repress apoptosis in microglial cells in the ischemic stroke cell model (Li and Sui, 2020). In MACO rats, valproate treatment represses ischemic stroke-induced disorders, including cell apoptosis and cerebral infarction (Li and Sui, 2020).

### Isosteviol Sodium

It is said that isosteviol sodium has neuroprotective effects and can block brain damage after ischemic stroke (Rösing et al., 2020). GAS5 over-expression diminishes miR-146a-5p to increase Notch1 expression, leading to promoting microglial M1 polarization and reducing microglial M2 polarization (Zhang et al., 2019a). Isosteviol sodium increases miR-146a-5p expression and declines the expression of GAS5 and Notch1. Functionally, isosteviol sodium enhances microglia M2 polarization through the GAS5-miR-146a-5p-Notch1 axis, which can ultimately attenuate brain injury caused by ischemic stroke in mice (Zhang et al., 2019a).

### Sulfasalazine

Sulfasalazine is a prodrug used for the treatment of autoimmune diseases, including rheumatoid arthritis and ulcerative colitis (Choi and Fenando, 2021). HOTAIR serves as a molecular sponge for miR-136-5p to promote AKT2 expression. Down-regulated HOTAIR suppresses the AKT2-NF-κB axis via increasing miR-136-5p (Duan et al., 2018). Sulfasalazine can decrease HOTAIR expression by inhibiting c-Myc in microglia. Sulfasalazine represses the microglial M1-like phenotype through the HOTAIR-miR-136-5p-AKT2-NF-κB axis, contributing to suppressing demyelination (Duan et al., 2018).

## Discussion and Conclusion

Microglia and astrocytes are glial cells that maintain tissue homeostasis in the nervous system. Accumulating evidence reports that both microglia and astrocytes have pro-inflammatory phenotype (M1/A1) and anti-inflammatory phenotype (M2/A2). Activated microglia and activated astrocytes can lead to inflammation, and the inflammation can contribute to microglial injuries, astrocyte injuries, and neuron damages. Inflammatory conditions also accelerate disease progression. LncRNAs and circRNAs are two ncRNAs involved in neural cell behaviors and nerve system development. For instance, up-regulated H19 increases CCL2 expression via sponging miR-1-3p, which can enhance astrocyte proliferation (Li P. et al., 2020). Knockdown of circHIPK2 can enhance NSC differentiation into neurons and promote neuronal plasticity (Wang et al., 2020a). LncRNA BDNF-antisense (BDNF-AS) is increased in the human amygdala of alcohol use disorder patients who started to drink before 21 years old. Up-regulated BDNF-AS reduces BDNF signaling, resulting in inhibiting synaptic plasticity in the amygdala (Bohnsack et al., 2019). Xu K. et al. (2020) proved that circGRIA1 deficiency contributes to the improvement of synaptogenesis and synaptic plasticity. LncRNA AtLAS is down-regulated in the dorsal medial prefrontal cortex in dominant mice. AtLAS can increase synapsin 2b (syn2b) expression. Syn2b binds with a-amino-3-hydroxy-5-methyl-4-isoxazolepropionic acid receptors (AMPAR) to restrain AMPAR membrane expression. Therefore, syn2b reduces AMPAR-correlated synaptic transmission. Decreased AtLAS contributes to enhanced synaptic strength in dominant mice (Ma et al., 2020).

**FIGURE 1:**
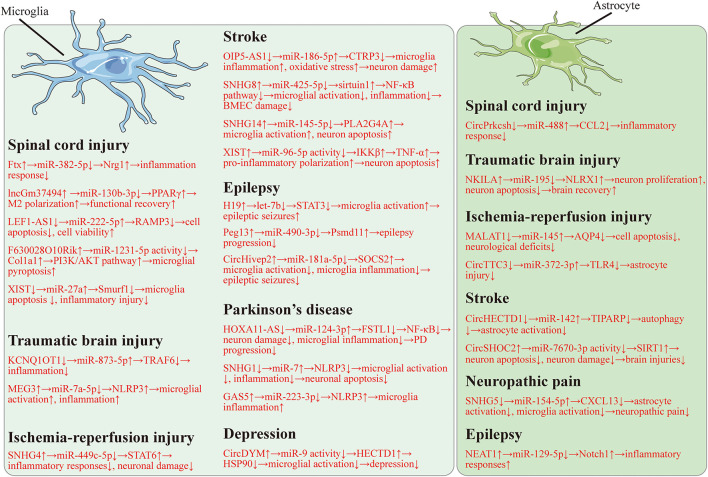
The lncRNA/circRNA-miRNA-mRNA regulatory network in microglia and astrocyte regulated neurological diseases.

Here, we found that lncRNAs and circRNAs can display positive or negative roles in microglia (Table 1) and astrocyte (Table 2) mediated neurological diseases. And the regulatory mechanisms of lncRNAs and circRNAs are related to microglia dysfunction, astrocyte dysfunction, neuron damage, and inflammation. It is widely indicated that both lncRNAs and circRNAs can serve as ceRNAs to inhibit miRNA activity. In this review, the lncRNA/circRNA-miRNA-mRNA regulatory network also exists in microglia and astrocyte mediated neurological diseases, including SCI (Figure 1; Shao et al., 2020; Xu S. et al., 2020; Zhao Q. et al., 2020; Cui et al., 2021; Xiang et al., 2021; Chen et al., 2022), TBI (He et al., 2021; Liu N. et al., 2021; Meng et al., 2021), cerebral IRI (Figure 1; Wang et al., 2020c; Zhang S. et al., 2020; Yang et al., 2021), stroke (Figure 1; Qi et al., 2017; Han B. et al., 2018; Chen W. et al., 2020; Chen Y. et al., 2021; Tian et al., 2021; Zhang M. et al., 2021), epilepsy (Figure 1; Feng et al., 2020; Han C. L. et al., 2020; Wan and Yang, 2020; Xiaoying et al., 2020), neuropathic pain (Figure 1; Chen M. et al., 2020), PD (Figure 1; Cao et al., 2018, 2021; Xu W. et al., 2020), and depression (Figure 1; Zhang Y. et al., 2020). We also found that some therapies can impair the ncRNA-glial cell axis and thus inhibit disease development. DEX can suppress SNHG14 to attenuate microglial activation, thus, to repress IRI (Ta Na et al., 2020). Valproate can decline RMRP to decrease microglia injuries. Isosteviol sodium promotes microglia M2 polarization via suppressing GAS5. Valproate and isosteviol sodium eventually hinder ischemic stroke (Zhang et al., 2019a; Li and Sui, 2020). Sulfasalazine reduces HOTAIR expression and therefore represses the microglial M1-like phenotype, which can finally suppress demyelination (Duan et al., 2018). These results suggest a promising treatment strategy based on the ncRNA-glial cell axis for neurological diseases. In this review, we collected evidence to reveal the relationships between lncRNAs/circRNAs and neurological diseases. We hope that this review will provide new sights for more clinical therapies, thereby allowing us to treat neurological diseases more effectively.

**TABLE 1:** LncRNAs and circRNAs in microglia mediated neurological diseases.

Nc-RNA	Disease	Expression	Functions	Role	References
Ftx	SCI	Down	Ftx↑→miR-382-5p↓→Nrg1↑→inflammation response↓	Suppressor	Xiang et al., 2021
lncGm37494	SCI	Up	lncGm37494↑→miR-130b-3p↓→PPARγ↑→M2 polarization↑→functional recovery↑	Suppressor	Shao et al., 2020
LEF1-AS1	SCI	Up	LEF1-AS1↓→miR-222-5p↑→RAMP3↓→cell apoptosis↓, cell viability↑	Promoter	Cui et al., 2021
F630028O10Rik	SCI	Up	F630028O10Rik↑→miR-1231-5p activity↓→Col1a1↑→PI3K/AKT pathway↑→microglial pyroptosis↑	Promoter	Xu K. et al., 2020
XIST	SCI	Up	XIST↓→miR-27a↑→Smurf1↓→microglia apoptosis ↓, inflammatory injury↓	Promoter	Zhao Y. et al., 2020
MALAT1	SCI	Up	MALAT1↑→miR-199b↓→IKKβ/NF-κB signal↑→inflammatory responses↑	Promoter	Zhou et al., 2018
KCNQ1OT1	TBI	Up	KCNQ1OT1↓→miR-873-5p↑→TRAF6↓→inflammation↓	Promoter	Liu D. et al., 2021
HOTAIR	TBI	Up	HOTAIR↓→MYD88↓→microglial activation↓, inflammatory factor release↓→TBI↓	Promoter	Cheng et al., 2021
MEG3	TBI	Up	MEG3↑→miR-7a-5p↓→NLRP3↑→microglial activation↑, inflammation↓	Promoter	Meng et al., 2021
MEG3	IRI	Up	MEG3↓→KLF4↑→M1 polarization↓, M2 polarization↑→neuroinflammation↓→cerebral IRI↓	Promoter	Li T. et al., 2020
SNHG4	IRI	Down	SNHG4↑→miR-449c-5p↓→STAT6↑→inflammatory responses↓, neuronal damage↓	Suppressor	Zhang N. et al., 2020
SNHG3	IRI	Up	SNHG3↓→HDAC3↓→microglial activation↓, inflammatory factor release↓	Promoter	Huang D. et al., 2021
MALAT1	IRI	Up	MALAT1↑→MyD88/IRAK1/TRAF6 signal↑→inflammatory response↑→cerebral IRI↓	Promoter	Wang and Zhou, 2018
NEAT1	IRI	Up	NEAT1↓→M1 polarization↓→neuronal apoptosis↓	Promoter	Ni et al., 2020
U90926	Stroke	Up	U90926↓→CXCL2↓→neutrophil infiltration↑→brain injury↑	Promoter	Chen J. et al., 2021
OIP5-AS1	Stroke	Down	OIP5-AS1↓→miR-186-5p↑→CTRP3↓→microglia inflammation↑, oxidative stress↑→neuron damage→	Suppressor	Chen Y. et al., 2021
lincRNA-EPS	Stroke	Up	lincRNA-EPS→microglia inflammation↓→neuron regeneration↑	Suppressor	Zhang B. et al., 2020
SNHG8	Stroke	Down	SNHG8↑→miR-425-5p↓→sirtuin1↑→NF-κB pathway↓→microglial activation↓, inflammation↓?BMEC damage↓	Suppressor	Tian et al., 2021
H19	Stroke	Up	H19↓→HDAC1↓→M2 polarization↑→neuroinflammation↓	Promoter	Wang et al., 2017
Nespas	Stroke	Up	Nespas↑→TAK1↓→NF-κB↓→microglia death↓, neuroinflammation↓	Suppressor	Deng et al., 2019
1810034E14Rik	Stroke	Down	1810034E14Rik↑→microglia activation↓, inflammation↓, neuronal damage↓	Suppressor	Zhang et al., 2019a
SNHG14	Stroke	Up	SNHG14↑→miR-145-5p↓→PLA2G4A↑→microglia activation↑, neuron apoptosis↑	Promoter	Qi et al., 2017
XIST	Stroke	Up	XIST↑→miR-96-5p activity↓→IKKβ↑→TNF-α↑→pro-inflammatory polarization↑→neuron apoptosis↑	Promoter	Zhang M. et al., 2021
Lncenc1	Neuropathic pain	Up	Lncenc1↑→EZH2↑→BAI1↓→microglia activation↑, inflammatory response↑	Promoter	Zhang Z. et al., 2021
H19	Epilepsy	Up	H19↑→JAK/STAT3 pathway↑→microglia activation↑, astrocyte activation↑	Promoter	Han C. L. et al., 2018
			H19↑→let-7b↓→STAT3↓→microglia activation→, astrocyte activation↑→epileptic seizures↑	Promoter	Han T. S. et al., 2020
Peg13	Epilepsy	Down	Peg13↑→miR-490-3p↓→Psmd11↑→epilepsy progression↓	Suppressor	Feng et al., 2020
uc.80-	Depression	Down	uc.80-↑→M1 polarization↓, M2 polarization↑, neuron apoptosis↓→depression↓	Suppressor	Gu et al., 2020
HOXA11-AS	PD	Up	HOXA11-AS↓→miR-124-3p↑→FSTL1↓→NF-κB↓→neuron damage↓, microglial inflammation↓→PD progression↓	Promoter	Cao et al., 2021
MALAT1	PD	Up	MALAT1↓→NRF2↑→microglial inflammation↓→neuron damage↓	Promoter	Cai et al., 2020b
lincRNA-p21	PD	Up	p53→lincRNA-p21↑→PKC-δ↑→microglia activation↑, inflammation↑→neuron degeneration↑	Promoter	Ye et al., 2018
SNHG1	PD	Up	SNHG1↓→miR-7↑→NLRP3↓→microglial activation↓, inflammation↓→neuronal apoptosis↓	Promoter	Cao et al., 2018
GAS5	PD	Up	GAS5↑→miR-223-3p↓→NLRP3↑→microglia inflammation↑	Promoter	Xu S. et al., 2020
GAS5	MS	Up	GAS5↑→IRF4↓→M2 polarization↓→myelin loss↑	Promoter	Sun et al., 2017
CircHivep2	Epilepsy	Down	CircHivep2↑→miR-181a-5p↓→SOCS2↑→microglia activation↓, microglia inflammation↓→epileptic seizures↓	Suppressor	Xiaoying et al., 2020
CircDYM	Depression	Down	CircDYM↑→miR-9 activity↓→HECTD1↑→HSP90↓→microglial activation↓→depression↓	Suppressor	Zhang S. et al., 2020

*ncRNA, non-coding RNA; SCI, spinal cord injury; Nrg1, Neuregulin-1; PPARγ, peroxisome proliferator-activated receptor γ; LEF1-AS1, lymphoid enhancer-binding factor 1 (LEF1) antisense RNA 1; PI3K, phosphoinositide 3-kinase; AKT, protein kinase B; XIST, X-inactive specific transcript; Smurf1, smad ubiquitination regulatory factor 1; MALAT1, metastasis-associated lung adenocarcinoma transcript 1; IKKβ, IkappaB kinase β; NF-κB, nuclear factor kappa B; TBI, traumatic brain injury; KCNQ1OT1, KCNQ1 overlapping transcript 1; TRAF6, tumor necrosis factor receptor-related factor 6; HOTAIR, HOX antisense intergenic RNA; MYD88, myeloid differentiation factor-88 adaptor protein; MEG3, maternally expressed gene 3; IRI, ischemia-reperfusion injury; KLF4, Krüppel-like factor 4; SNHG4, small nucleolar RNA host gene 4; STAT6, signal transducers and activators of transcription 6; HDAC3, histone deacetylase 3; IRAK1, IL-1 receptor-associated kinase 1; NEAT1, nuclear enriched abundant transcript 1; CXCL2, C-X-C motif ligand 2; OIP5-AS1, opa-interacting protein 5 antisense RNA 1; CTRP3, C1q/tumor necrosis factor-related protein 3; BMEC, brain microvascular endothelial cell; TAK1, transforming growth factor-beta-activated kinase 1; PLA2G4A, cytosolic phospholipase A2 (cPLA2) group IVA; TNF-α, tumor necrosis factor α; Lncenc1, lncRNA embryonic stem cells expressed 1; EZH2, enhancer of zeste homolog 2; BAI1, brain-specific angiogenesis inhibitor 1; JAK, Janus kinase; Psmd11, 26S proteasome non-ATPase regulatory subunit 11; PD, Parkinson’s disease; FSTL1, follistatin-like 1; NRF2, nuclear factor (erythroid-derived 2)-like-2 factor; PKC-δ, protein kinase C δ; NLRP3, nod-like receptor protein 3; GAS5, growth arrest-specific 5; SOCS2, suppressor of cytokine signaling-2; MS, multiple sclerosis; IRF4, interferon-regulatory factor 4; circDYM, circRNA DYM; HECTD1, HECT domain E3 ubiquitin protein ligase 1; HSP90, 90-kDa heat shock protein.*

**TABLE 2:** LncRNA and circRNAs in astrocyte mediated neurological diseases.

NcRNA	Disease	Expression	Functions	Role	References
SNHG5	SCI	Up	SNHG5↑→KLF4↓→cell viability↑, apoptosis↓→SCI progression?	Promoter	Jiang and Zhang, 2018
NKILA	TBI	Up	NKILA↑→miR-195↓→NLRX1↑→neuron proliferation↑, neuron apoptosis↓→brain recovery↑	Suppressor	He et al., 2021
MALAT1	TBI	Down	MALAT1↑→AQP4↓, NF−κB↓, IL-6↓→astrocyte swelling↓→brain edema↓	Suppressor	Zhang et al., 2019c
MALAT1	IRI	Up	MALAT1↓→miR-145↑→AQP4↓→cell apoptosis↓, neurological deficits↓	Promoter	Wang et al., 2020b
TUG1	IRI	Up	TUG1↓→miR-142-3p↑→cell viability↑, cell apoptosis↓	Promoter	Li et al., 2021
SNHG5	Neuropathic pain	Up	SNHG5↓→miR-154-5p↑→CXCL13↓→astrocyte activation↓, microglia activation↓→neuropathic pain↓	Promoter	Chen M. et al., 2020
CASC2	Epilepsy	Down	CASC2↑→PTEN↑→astrocyte activation↓, adenosine metabolism↓	Suppressor	Zhu et al., 2020
UCA1	Epilepsy	Down	UCA1↑→astrocyte activation↓, GLAST↓	Suppressor	Wang et al., 2020a
			UCA1↑→MEF2C↑→NF-κB signal↓→astrocyte inflammation↓→epilepsy↓	Suppressor	Yu et al., 2020
NEAT1	Epilepsy	Up	NEAT1↑→miR-129-5p↓→Notch1↑→inflammatory responses↑→	Promoter	Wan and Yang, 2020
PVT1	Epilepsy	Up	PVT1↓→Wnt pathway↓→BDNF↑, neuronal loss↓, astrocyte activation↓, pro-inflammatory cytokine↓	Promoter	Zhao et al., 2019
Gm14205	Depression	Up	Gm14205↑→oxytocin receptors↓→NLRP3 inflammasome activation↑→PPD↑	Promoter	Zhu and Tang, 2020
Gm13568	MS	Up	IL-9→Gm13568↑→Notch1/STAT3 pathway↑→astrocyte inflammation↑→EAE process↑	Promoter	Liu N. et al., 2021
MEG3	AD	Down	MEG3↑→PI3K/AKT pathway↓→astrocyte activation↓, neuronal damage↓, cognitive impairment↓	Suppressor	Yi et al., 2019
CircPrkcsh	SCI	UP	CircPrkcsh↓→miR-488↑→CCL2↓→inflammatory response↓	Promoter	Chen et al., 2022
CircTTC3	IRI	Up	CircTTC3↓→miR-372-3p↑→TLR4↓→astrocyte injury↓, NSC proliferation→, NSC differentiation↑	Promoter	Yang et al., 2021
CircHECTD1	Stroke	Up	CircHECTD1↓→miR-142 activity↑→TIPARP↓→autophagy↓→astrocyte activation↓	Promoter	Han B. et al., 2018
CircSHOC2	Stroke	Up	CircSHOC2↑→miR-7670-3p activity↓→SIRT1↑→neuron apoptosis↓, neuron damage↓→brain injuries↓	Suppressor	Chen W. et al., 2020
CircIgf1r	Epilepsy	Up	CircIgf1r↓→autophagy↑→A2 polarization↑→neuronal toxicity↓	Promoter	Shao et al., 2021
CircSTAG1	Depression	Down	CircSTAG1↑→FAAH↓→astrocyte dysfunction↓→depressive-like behaviors↓	Suppressor	Huang et al., 2020
CircHIPK2	Depression	Up	CircHIPK2↓→astrocyte dysfunction↓→depression↓	Promoter	Zhang et al., 2019b

*ncRNA, non-coding RNA; SNHG5, small nucleolar RNA host gene 5; SCI, spinal cord injury; KLF4, Krüppel-like factor 4; NKILA, nuclear transcription factor NF-κB interacting lncRNA; TBI, traumatic brain injury; NLRX1, nucleotide-binding leucine-rich repeat containing family member X1; MALAT1, metastasis-associated lung adenocarcinoma transcript 1; AQP4, aquaporin 4; NF-κB, nuclear factor kappa B; IL-6, interleukin-6; IRI, ischemia-reperfusion injury; TUG1, taurine upregulated gene 1; CXCL13, C-X-C motif ligand 13; CASC2, cancer susceptibility candidate 2; PTEN, phosphatase and tensin homolog; GLAST, glutamate aspartate transporter; MEF2C, myocyte enhancer factor 2C; NEAT1, nuclear enriched abundant transcript 1; PVT1, plasmacytoma variant translocation 1; BDNF, brain-derived neurotrophic factor; NLRP3, nod-like receptor protein 3; PPD, postpartum depression; MS, multiple sclerosis; STAT3, signal transducers and activators of transcription 3; EAE, experimental autoimmune encephalomyelitis; MEG3, maternally expressed gene 3; AD, Alzheimer’s disease; PI3K, phosphoinositide 3-kinase; AKT, protein kinase B; CCL2, chemokine CC motif ligand 2; circTTC3, circRNA TTC3; TLR4, Toll-like receptor 4; NSC, neural stem cell; TIPARP, TCDD inducible poly(ADP-ribose) polymerase; SIRT1, sirtuin 1; FAAH, fatty acid amide hydrolase.*

**FIGURE 2:**
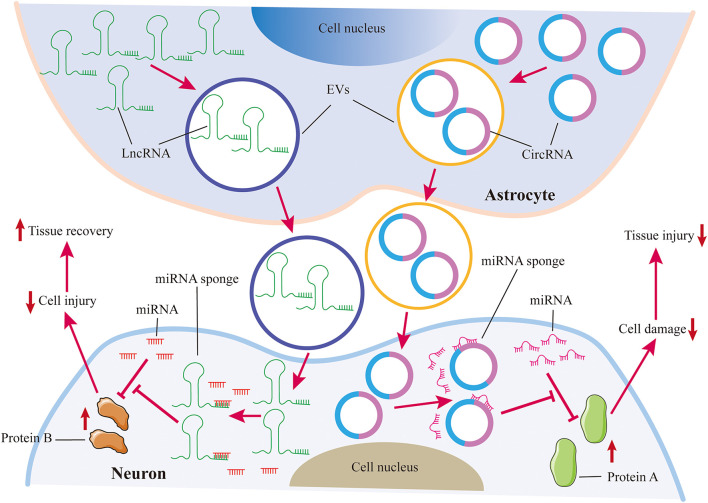
Astrocytes protect neurons against injuries via EVs. Astrocytes transfer lncRNAs and circRNAs to neurons through EVs. Both lncRNAs and circRNAs can act as miRNA sponges to inhibit miRNA repression on target proteins, which can restrain neuron damage. LncRNAs and circRNAs from astrocyte-derived EVs are beneficial for tissue repair in the nervous system.

Recently, increasing evidence reveals that lncRNAs and circRNAs can be transferred into recipient cells via EVs and control disease development. For example, astrocytes can transfer lncRNAs and circRNAs to neurons through EVs and repress neuron injuries, leading to alleviating nervous system damage (Figure 2; Chen W. et al., 2020; He et al., 2021). Shao et al. (2020) found that exosomal lncGm37494 from ADSCs stimulates microglia M2 polarization to enhance functional recovery after SCI. One study reported that intranasally delivered lincRNA-Cox2-siRNA loaded EVs can suppress microglia proliferation upon LPS treatment in mice (Liao et al., 2020). And Zhang et al. investigated that lincRNA-EPS can be loaded in biomimetic vesicles targeting microglia. And lincRNA-EPS-loaded biomimetic vesicles abolish microglial inflammation to promote neuron regeneration *in vivo* (Zhang B. et al., 2020). These suggest that EV-loaded ncRNAs targeted glial cells could be promising therapeutic therapies for the treatment of neurological disorders.

Presently, despite the great progress that has been made in neurodegeneration, the researches based on the ncRNA-glial cell axis are still lacking. It is widely suggested that LPS-induced neuroinflammation can mimic the microenvironment in neurodegenerative diseases (Hu et al., 2018). LPS-treated models may be useful to explore the pathological mechanism in neurodegeneration.

## Author Contributions

All authors contributed to the study conception and design, read, and agreed to the published version of the manuscript.

## Conflict of Interest

The authors declare that the research was conducted in the absence of any commercial or financial relationships that could be construed as a potential conflict of interest.

## Publisher’s Note

All claims expressed in this article are solely those of the authors and do not necessarily represent those of their affiliated organizations, or those of the publisher, the editors and the reviewers. Any product that may be evaluated in this article, or claim that may be made by its manufacturer, is not guaranteed or endorsed by the publisher.
